# Spiking network simulation code for petascale computers

**DOI:** 10.3389/fninf.2014.00078

**Published:** 2014-10-10

**Authors:** Susanne Kunkel, Maximilian Schmidt, Jochen M. Eppler, Hans E. Plesser, Gen Masumoto, Jun Igarashi, Shin Ishii, Tomoki Fukai, Abigail Morrison, Markus Diesmann, Moritz Helias

**Affiliations:** ^1^Simulation Laboratory Neuroscience – Bernstein Facility for Simulation and Database Technology, Institute for Advanced Simulation, Jülich Aachen Research Alliance, Jülich Research CentreJülich, Germany; ^2^Programming Environment Research Team, RIKEN Advanced Institute for Computational ScienceKobe, Japan; ^3^Institute of Neuroscience and Medicine (INM-6), Institute for Advanced Simulation (IAS-6), Jülich Research Centre and JARAJülich, Germany; ^4^Department of Mathematical Sciences and Technology, Norwegian University of Life SciencesAas, Norway; ^5^Advanced Center for Computing and Communication, RIKENWako, Japan; ^6^Neural Computation Unit, Okinawa Institute of Science and TechnologyOkinawa, Japan; ^7^Laboratory for Neural Circuit Theory, RIKEN Brain Science InstituteWako, Japan; ^8^Integrated Systems Biology Laboratory, Department of Systems Science, Graduate School of Informatics, Kyoto UniversityKyoto, Japan; ^9^Faculty of Psychology, Institute of Cognitive Neuroscience, Ruhr-University BochumBochum, Germany; ^10^Medical Faculty, RWTH UniversityAachen, Germany

**Keywords:** supercomputer, large-scale simulation, parallel computing, computational neuroscience, memory footprint, memory management, metaprogramming

## Abstract

Brain-scale networks exhibit a breathtaking heterogeneity in the dynamical properties and parameters of their constituents. At cellular resolution, the entities of theory are neurons and synapses and over the past decade researchers have learned to manage the heterogeneity of neurons and synapses with efficient data structures. Already early parallel simulation codes stored synapses in a distributed fashion such that a synapse solely consumes memory on the compute node harboring the target neuron. As petaflop computers with some 100,000 nodes become increasingly available for neuroscience, new challenges arise for neuronal network simulation software: Each neuron contacts on the order of 10,000 other neurons and thus has targets only on a fraction of all compute nodes; furthermore, for any given source neuron, at most a single synapse is typically created on any compute node. From the viewpoint of an individual compute node, the heterogeneity in the synaptic target lists thus collapses along two dimensions: the dimension of the types of synapses and the dimension of the number of synapses of a given type. Here we present a data structure taking advantage of this double collapse using metaprogramming techniques. After introducing the relevant scaling scenario for brain-scale simulations, we quantitatively discuss the performance on two supercomputers. We show that the novel architecture scales to the largest petascale supercomputers available today.

## 1. Introduction

In the past decade, major advances have been made that allow the routine simulation of spiking neuronal networks on the scale of the local cortical volume, i.e., containing up to 10^5^ neurons and 10^9^ synapses, including the exploitation of distributed and concurrent computing, the incorporation of experimentally observed phenomena such as plasticity, and the provision of appropriate high-level user interfaces (Davison et al., 2008; Bednar, 2009; Eppler et al., 2009; Hines et al., 2009; Goodman and Brette, 2013; Zaytsev and Morrison, 2014). However, such models are intrinsically limited. Firstly, a cortical neuron receives only about 50% of its synaptic inputs from neurons in the same cortical volume; in such models, the other half is generally abstracted as a random or constant input. Consequently, larger models are required to arrive at self-consistent descriptions. Secondly, many cognitive tasks involve the co-ordinated activity of multiple brain areas. Thus, in order to understand such processes, it is necessary to develop and investigate models on the brain scale.

In a previous study (Kunkel et al., 2012b), we presented data structures that allow the neuronal network simulator NEST (Gewaltig and Diesmann, 2007) to exploit the increasingly available supercomputers such as JUQUEEN and the K computer (Helias et al., 2012). Although we could carry out benchmarks utilizing over 100,000 cores, analysis of the memory consumption (Section 3.1) reveals that at such large sizes, the infrastructure required on each machine to store synapses with local targets becomes the dominant component.

**Figure 1:**
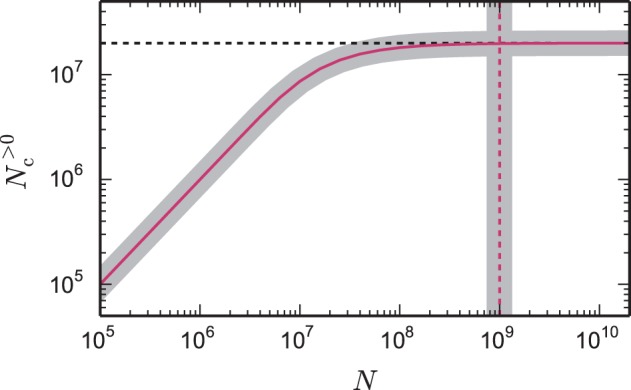
**Number of local target lists approaches the number of local synapses**. The gray curve shows the expected number of target lists per core *N*^>0^_c_, given by Equation (6), that contain at least one synapse as a function of the total network size *N*. The pink curve (1 − exp(− *K*_VP_/*N*))*N* is an approximation (7) for large networks. Here, each core represents *N*_VP_ = 2000 neurons with *K* = 10,000 synapses per neuron, which is a realistic example. In the limit of large *N* the number of target lists approaches the number of local synapses *K*_VP_ = *K*_VP_ (black dashed horizontal line). The gray vertical line marks the network size *N*_ζ_, given by Equation (9), at which the number of target lists has reached ζ = 99% of the limit *K*_VP_. The pink dashed vertical line at *K*_VP_/2/(1 − ζ) is an approximation (10) of the full expression (9).

The reason can be understood with a simple calculation. Assuming each neuron contacts 10,000 other neurons, and that these neurons are randomly distributed over the entire supercomputer, then on a system with 100,000 cores, the probability of a core containing more than one target neuron is rather small, and the majority of cores will contain no target neurons of the given neuron. This is illustrated in Figure 1; as the total network size increases (whilst maintaining a constant number of neurons on a core), the number of target lists of non-zero length approaches the number of synapses on the local core and, consequently, each target list has an expected length of 1. As each new target list comes with a certain memory overhead, the average total costs per synapse increase with increasing network size. This acceleration in memory consumption only stops when each target list is either of length 0 or length 1 and from then on each synapse carries the full overhead of one target list. With realistic parameters, the largest networks that can be represented on supercomputers using the technology employed in Helias et al. (2012) reach this limit.

The memory model introduced in Kunkel et al. (2012b) triggered the major advance of using sparse tables to store incoming connections, thus reducing the memory overhead for target lists of length 0. However, the memory overhead for neurons with local targets is still substantial, as the data structure is designed to enable variable amounts of heterogeneous connection types, e.g., static synapses and various types of plasticity including short-term plasticity (Tsodyks et al., 1998, 2000) and spike-timing dependent plasticity (see e.g., (Morrison et al., 2008), for a review). Nevertheless, as the network size increases, it becomes increasingly common that a neuron has only one local target, thus the majority of this structure is redundant: both the number (just 1) and the type of the connection are known.

Thus, the challenge is to develop a data structure that is streamlined for the most common case (on large systems) that a neuron has one local target, and yet allows the full flexibility with respect to connection type and number when needed. A constraint on the granularity of the parallelization is the assumption that the neuron objects themselves are not distributed but viewed as atomistic and simulated on a single compute node as an entity. Consequently the connection infrastructure only needs to represent synaptic connections, not connections between different compartments of a neuron. In Section 3.2, we present a data structure that fulfills these criteria by self-collapsing along two dimensions: the heterogeneity collapses to a single well-defined connection type and the dynamic length of the connections vector collapses to a single element. Moreover, a redesign of the synapse data structure and the handshaking algorithm at connection time (see Section 3.3) allow the polymorphism of the synapse types to be exploited without requiring a pointer to a virtual function table, thus saving 8 B for every synapse in the network. Making use of the limited number of neurons local to a core further allows us to replace the full 8 B target pointer by a 2 B index and combining the delay and the synapse type into a joint data structure saves another 8 B. The creation of many small synapse objects presents a challenge to the memory allocation which we meet by implementing a dedicated pool allocator in Section 3.4. Finally, in Section 3.5 a new data structure to store local neurons is introduced. The sparse node array exploits the regularity in assigning neurons to machines and thereby eliminates memory overhead for non-local neurons at the expense of an increased number of search steps to locate a node. The major result of this study is that we are now capable of employing the full size of currently available petascale computers such as JUQUEEN and K.

In Section 2.1 we describe the basic characteristics of the software environment under study and Section 2.2 specifies the neuronal network model used to obtain quantitative data. Section 2.3 extends the mathematical memory model previously introduced in Kunkel et al. (2012b) which we use to analyze the scaling properties of alternative data structures.

In Section 3.1 we first investigate the memory consumption of NEST on petascale computers in detail. In the following sections we describe the data structures, corresponding algorithms, and the allocator outlined above. Finally in Section 3.6 we quantitatively compare the resulting new (4g) simulation code with the previous (3g) version on the two supercomputers JUQUEEN and K. The capability of the fourth generation code is demonstrated by orchestrating all the available main memory of the K computer in a single simulation. In the concluding section we assess our achievements in terms of flexibility and network size in the light of previous work and discuss limitations.

This article concludes a co-development project for the K computer in Kobe, which started in 2008 (Diesmann, 2013). Preliminary results have been published in abstract form (Diesmann, 2012; Kunkel et al., 2013) and as a joint press release of the Jülich Research Centre and RIKEN (RIKEN BSI, 2013). The conceptual and algorithmic work described here is a module in our long-term collaborative project to provide the technology for neural systems simulations (Gewaltig and Diesmann, 2007).

## 2. Materials and methods

### 2.1. NEST simulator

NEST is a simulation software for spiking neuronal networks of single- and few-compartment neuron models (Gewaltig and Diesmann, 2007). It incorporates a number of technologies for the accurate and efficient simulation of neuronal systems such as the exact integration of models with linear subthreshold dynamics (Rotter and Diesmann, 1999), algorithms for synaptic plasticity (Morrison et al., 2007, 2008; Potjans et al., 2010), the framework for off-grid spiking including synaptic delays (Morrison et al., 2005a; Hanuschkin et al., 2010) and the Python-based user interface PyNEST/CyNEST (Eppler et al., 2009; Zaytsev and Morrison, 2014). NEST is developed by the NEST Initiative and available under the GNU General Public License. It can be downloaded from the website of the NEST Simulator (http://www.nest-simulator.org).

NEST uses a hybrid parallelization strategy during the setup and simulation phase with one MPI process per compute node and multi-threading based on OpenMP within each process. The use of threads instead of MPI processes on the cores is the basis of light-weight parallelization, because process-based distribution employed in MPI enforces the replication of the entire application on each MPI process and process management entails additional overhead (Eppler et al., 2007; Plesser et al., 2007). Thread-parallel components do not require the duplication of data structures *per se*. However, the current implementation of NEST duplicates parts of the connection infrastructure for each thread to achieve good cache performance during the thread-parallel delivery of spike events to the target neurons.

Furthermore, on a given supercomputer the number of MPI processes may have limits; on K, for example, there can be only one MPI job per node and the total number of MPI jobs is limited to 88,128. The neurons of the network are evenly distributed over the compute nodes in a round-robin fashion and communication between machines is performed by collective MPI functions (Eppler et al., 2007). The delivery of a spike event from a given neuron to its targets requires that each receiving machine has the information available to determine whether the sending neuron has any targets local to that machine. In NEST, this is realized by storing the outgoing synapses to local targets in a data structure logically forming a target list. For the 3rd generation kernel, this data structure is described in detail in Kunkel et al. (2012b) and for the 4th generation kernel in Section 3.2. For comparison, the two data structures are illustrated in Figure **3**. In addition, the memory consumption caused by the currently employed collective data exchange scheme (MPI_Allgather) increases with the number of MPI processes.

### 2.2. Network model

All measurements of memory usage and run time are carried out for a balanced random network model (Brunel, 2000) of 80% excitatory and 20% inhibitory integrate-and-fire neurons with alpha-shaped post-synaptic currents. Both types of neurons are represented by the NEST model iaf_neuron with a homogeneous set of parameters. All excitatory-excitatory connections exhibit spike-timing dependent plasticity (STDP) and all other connections are static. Simulations performed with the 3rd generation simulation kernel (Helias et al., 2012; Kunkel et al., 2012b) employ the models stdp_pl_synapse_hom and static_synapse whereas simulations run with the 4th generation simulation kernel presented in this manuscript use the novel high-performance computing (HPC) versions of these models (stdp_pl_synapse_hom_hpc and static_synapse_hpc, described in Section 3.3). We use two sets of parameters for the benchmarks. Within each set, the only parameter varied is the network size in terms of number of neurons *N*.

**Set 1** The total number of incoming connections per neuron is fixed at *K* = 11,250 (9000 excitatory, 2250 inhibitory). The initial membrane potentials are drawn from a normal distribution with μ = 9.5 mV and σ = 5.0 mV. The initial synaptic weights are set to *J*_*E*_ = 45.61 pA for excitatory and to *J*_*I*_ = −*gJ*_*E*_, *g* = 5 for inhibitory synapses. All neurons receive excitatory external Poissonian input causing a mean membrane potential of η $V_{\text{th}}=\tau_{\text{syn}}J_{E}\frac{\tau_{m}}{C_{m}}\nu_{\text{ext}.}$. With η = 1.685, *V*_th_ = 20 mV, τ_*m*_ = 10 ms, *C*_*m*_ = 250 pF, and τ_syn_ = 0.3258 ms, this corresponds to the input spike rate of $\nu_{\text{ext}.}=\eta \frac{V_{\text{th}}}{\tau_{\text{syn}}J_{E}\frac{\tau_{m}}{C_{m}}}≃20,856$ spikes per second summed over all external inputs to a neuron. Within the simulation period of 1 s, each neuron fires on average 7.6 times. Spikes are communicated every 1.5 ms, corresponding to the synaptic delay, but neuronal state variables are advanced in steps of 0.1 ms. For further details of the network model such as neuronal and synaptic parameters please see the example script hpc_benchmark.sli, which is available in the next major release of NEST.
**Set 2** For the second set of benchmarks, the number of incoming connections per neurons is reduced to *K* = 6000. The other parameters are adapted to obtain an irregular activity state with an average rate of 4.5 spikes per second. The adapted parameters are *J*_*E*_ = 50 pA, *g* = 7, and η = 1.2. All other parameters are the same as in set 1.

### 2.3. Memory-usage model

Our efforts to redesign the objects and fundamental data structures of NEST toward ever more scalable memory usage are guided by the method introduced in Kunkel et al. (2012b). The method is based on a model which describes the memory usage of a neuronal network simulator as a function of the network parameters, i.e., the number of neurons *N* and the number *K* of synapses per neuron as well as the parameters characterizing the distribution of the simulation code over the machine, i.e., the number of compute nodes *M* and the number of threads *T* running on each compute node. Threads are also termed “virtual processes” due to NEST's internal treatment of threads as if they were MPI processes by completely separating their memory areas. This replication of data structures for each thread is reflected in expressions of the memory consumption of the synaptic data structures that depend only on the product *MT*, as shown in Section 2.4. In the following, we therefore use the terms “the total number of virtual processes” synonymously with “the total number of threads,” both referring to *MT*. We apply the model to NEST to determine the data structures that dominate the total memory usage at a particular target regime of number of virtual processes. Once the critical parts of NEST have been identified, the model enables us to predict the effect of potential design changes for the entire range of the total number of threads from laptops to supercomputers. Furthermore, the model assists the benchmarking process as it facilitates the estimation of the maximum network size that just fits on a given number of compute nodes using a given number of threads each. We briefly restate the model here and describe the required alterations to the model for the petascale regime, which allow a more precise assessment of the contributions of different parts of infrastructure. For further details on the model and its practical application, please refer to our previous publications (Helias et al., 2012; Kunkel et al., 2012a,b).

Three main components contribute to the total memory consumption of a neuronal network simulator: the base memory usage of the simulator including external libraries such as MPI, 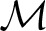
_0_ (*M*), the additional memory usage that accrues when neurons are created, 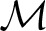
_n_ (*M*, *N*), and the additional memory usage that accrues when neurons are connected, 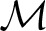
_c_ (*M*, *T*, *N*, *K*). The memory consumption per MPI process is given by

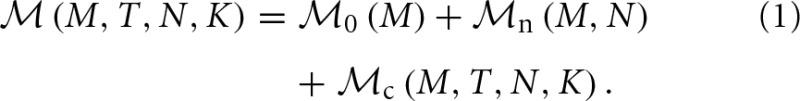



As suggested in Kunkel et al. (2012b), we determined 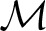
_0_ (*M*) by measuring the memory usage of NEST right after start-up, which was at most 268 MB on the K computer and 26 MB on JUQUEEN. However, in this study 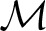
_0_ (*M*) also accounts for the communication buffer that each MPI process requires in order to receive spike information from other processes. As NEST uses MPI_Allgather to communicate spike data, the buffer grows proportionally with the number of MPI processes *M*. Hence, in the petascale regime the contribution of this buffer to the total memory usage is no longer negligible. Here, we assume that each MPI process maintains an outgoing buffer of size 1000, where each entry consumes 4 B, such that the memory that is taken up by the incoming buffer amounts to *M* × 4 kB. In NEST the communication buffers increase dynamically whenever the instantaneous rate of the simulated network requires more spikes to be communicated. In simulations of the benchmark network model described in Section 2.2 we measured send-buffer sizes of 568 entries (in a full K computer simulation), such that for this model the assumed buffer size of 1000 is a worst-case scenario.

Neuron objects in NEST are distributed across virtual processes in a round-robin fashion and connections are represented on the process of their post-synaptic neuron. We use the term “VP-local” to indicate that a neuron is local to a certain virtual process. As neurons with similar properties are typically created en bloc, the round-robin distribution scheme constitutes a simple form of static load-balancing for heterogeneous networks with varying numbers of incoming connections per neuron. If each virtual process owns sufficiently many neurons, the number of local connection objects is similar across processes. Therefore, in our model we let *N*_*M*_ = *N*/*M* and *K*_*M*_ = *N*_*M*_*K* denote the average number of neuron and connection objects per MPI process, and we let *N*_VP_ = *N*_*M*_/*T* and *K*_VP_ = *N*_VP_*K* denote the average number of VP-local neuron and connection objects.

In the regime of ~ 10,000 virtual processes, for a randomly connected network the targets of a neuron become more and more spread out. This results in the limiting case where *K* processes each own one of the targets and the remaining *MT* − *K* processes do not own any of the targets. As a consequence, the connection infrastructure becomes increasingly sparse, where the extent of sparseness can be quantified in a probabilistic way. Here we use the symbol ~ reading “on the order of” (Hardy and Wright, 1975, p. 7) in the physics sense (Jeffreys and Jeffreys, 1956, p. 23). This relation stating that two quantities are not differing by more than a factor of 10 needs to be distinguished from the big-O notation below (Section 2.4), which is used to describe the limit of a function.

To quantify the sparseness we define *p*_∅_ and *p*_1_ as the probabilities that a particular neuron has 0 or 1 local target on a given virtual process, respectively. Each neuron draws on average *K* source neurons from the set of *N* possible source neurons. If the incoming connections per neuron are drawn independently, on average *K*_VP_ source neurons are drawn on each virtual process. Due to the large numbers, the distribution around this mean value is narrow. The probability that a particular neuron is drawn as a source is 1/*N* and the probability that the neuron is not drawn as a source is 1 − 1/*N*. We can therefore adopt the simplifying assumption that *p*_∅_ = (1 − 1/*N*)^*K*_VP_^ expresses the average probability that a neuron does not connect to any VP-local target, such that *N*^∅^_c_ = *p*_∅_*N* denotes the expected number of neurons without any VP-local target. In this study we adapt the model to separately account for the neurons with exactly one VP-local target and for those with more than one VP-local target. We introduce *p*_1_ = (1 − 1/*N*)^*K*_VP_−1^ *K*_VP_/*N* as the average probability that a neuron has exactly one local target, such that *N*^1^_c_ = *p*_1_*N* denotes the expected number of neurons with only one local target. The remaining *N* − *N*^∅^_c_ − *N*^1^_c_ neurons connect to more than one VP-local target.

Throughout this study, we keep the average number of incoming connections per neuron fixed at either *K* = 11,250 or *K* = 6000 in accordance with the two employed benchmark network models (see Section 2.2), and we assume *T* = 8 threads per MPI process, which corresponds to the maximum number of threads per node supported on the K computer (see Section 2.5). We explicitly differentiate between connections with spike-timing dependent plasticity and connections with static weights. This is a trivial but useful extension to the model, which enables a more precise prediction of memory usage. For the case that all excitatory-excitatory connections exhibit STDP, the number of STDP connections per MPI process amounts to *K*^stdp^_*M*_ = *K*_*M*_β^2^, where β = 0.8 is the fraction of excitatory neurons, and the remaining *K*^stat^_*M*_ = *K*_*M*_ − *K*^stdp^_*M*_ synapses are static. In Helias et al. (2012), this differentiation between two connection types was not required as in NEST 2.2 (3g kernel) the employed models stdp_pl_synapse_hom and static_synapse have an identical memory usage of *m*^stdp^_c_ = *m*^stat^_c_ = 48 B.

With the above definitions, the memory consumption of the latter two terms of Equation (1) can be further decomposed into

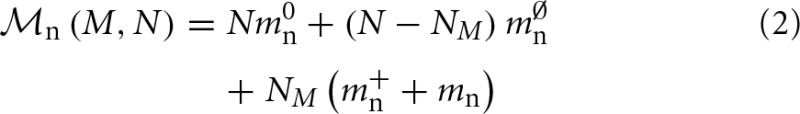

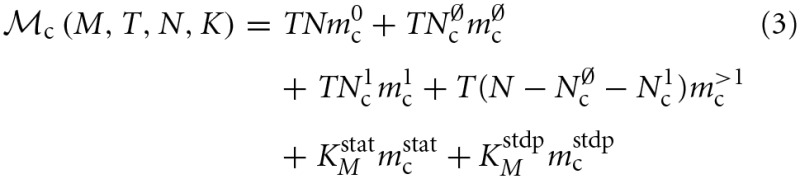





in order to capture the contributions of neuron and synapse objects and different parts of infrastructure. Table 1 summarizes the model parameters required to specify 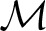
_n_ (*M*, *N*) and 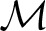
_c_ (*M*, *T*, *N*, *K*) and contrasts their values for the 3g (Helias et al., 2012) and 4g simulation technology. For convenience, we provide the values already at this point even though they are explained only in Section 3.

**Table 1:** **Parameter definitions and values of memory-usage model for 3g and 4g technology**.

	**Parameter**	**Description**	**Value in B**	
			**3g**	**4g**
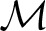 _n_ (*M*, *N*)	*m*_n_	memory usage of one neuron object of type iaf_psc_alpha	1100	
	*m*^0^_n_	memory overhead per neuron	0.33	0
	*m*^+^_n_	memory overhead per local neuron	16	24
	*m*^∅^_n_	memory overhead per non-local neuron	0	
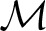 _c_ (*M*, *T*, *N*, *K*)	*m*^stat^_c_	memory usage of one connection object of type static_synapse (3g) or static_synapse_hpc (4g)	48	16
	*m*^stdp^_c_	memory usage of one connection object of type stdp_pl_synapse (3g) or stdp_pl_synapse_hpc (4g)	48	24
	*m*^0^_c_	memory overhead per neuron	0.33	
	*m*^1^_c_	memory overhead per neuron with one local target	96	24
	*m*^>1^_c_	memory overhead per neuron with more than one local target	160	128
	*m*^∅^_c_	memory overhead per neuron without local targets	0	

The top part of the table summarizes the parameters relevant for the memory consumption due to the neuronal infrastructure 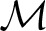
_#1_, the bottom part shows the parameters determining the memory consumption 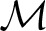
_c_ of the synaptic infrastructure. Lower case symbols *m* refer to the memory per object, where objects are neurons or connections, respectively. The columns “3g” and “4g” distinguish between the parameter values for the two kernel versions.

Note that we assume the same overhead *m*^>1^_*c*_ for all neurons with more than one local target, which means that we do not introduce any further distinction of possible synapse containers for the cases where more than one synapse needs to be stored (see Section 3.2 for the details). Here, we set *m*^>1^_*c*_ such that it corresponds to the most complex synapse container that can occur in simulations of the benchmark network model described in Section 2.2, which is a container that stores two different types of synapses in corresponding vectors. As a result of this worst-case assumption the model produces a slight overestimation of memory consumption.

Overall, however, the model underestimates the effectively required memory resources as the theoretically determined parameter values that we employ here reflect only the memory usage of the involved data types on a 64 bit architecture, but they cannot account for the memory allocation strategies of the involved dynamical data structures (Kunkel et al., 2012b).

### 2.4. Number and length of local target lists

Using the notation of Section 2.3 the probability of a neuron to be the source of a particular synapse is 1/*N* and consequently the probability of not being the source of any of the *K*_VP_ = *KN*/(*MT*) VP-local synapses is

(4) $$p_{\emptyset }={\left(1-\frac{1}{N}\right)}^{\frac{KN}{MT}}.$$

Empty target lists are not instantiated and therefore do not cause overhead by themselves. We recognize in Equation (4) the structure (1 + *x*/*N*)^*N*^ exposed by

$$p_{\emptyset }={\left[{\left(1-\frac{1}{N}\right)}^{N}\right]}^{\frac{K}{MT}}.$$

Thus, in the limit of large *N* we can use the definition of the exponential function lim_*N*→∞_ (1 + *x*/*N*)^*N*^ = exp(*x*) to replace the term [·]. Conceptually this corresponds to the approximation of the binomial probabilities $\left(\begin{array}{c} N\\ k \end{array}\right)p^{k}{\left(1-p\right)}^{N-k}$ by the corresponding Poisson probabilities $\frac{\lambda^{k}}{k!}\exp(-\lambda )$, where λ = *Np* = const. as *N* → ∞. In this limit we have

(5) $$\begin{array}{l} {\tilde{p}}_{\emptyset }=e^{-\frac{K}{MT}}\\ \;≃1-\frac{K}{MT}+\frac{1}{2}{\left(\frac{K}{MT}\right)}^{2}+O\left[{\left(\frac{K}{MT}\right)}^{3}\right], \end{array}$$

where in the second line we expanded the expression up to second order in the ratio $\frac{K}{MT}$ ≪ 1. We here use the big-O notation in the sense of infinitesimal asymptotics, which means that $O\left[{\left(\frac{K}{MT}\right)}^{3}\right]$ collects all terms of the form $f\left(\frac{K}{MT}\right)$ such that for any small $\frac{K}{MT}$ there exists a constant *C* fulfilling the relation $|f\left(\frac{K}{MT}\right)|\;<C{\left(\frac{K}{MT}\right)}^{3}$ as $\frac{K}{MT}$ → 0. In complexity theory big-O often implicitly denotes the infinite asymptotics when it refers to an integer variable *n*. Both use cases of the notation are intended and comply with its definition (Knuth, 1997, section 1.2.11.1).

The expected number of target lists with at least one synapse is

(6) $$\begin{array}{l} N_{\text{c}}^{>0}=(1-p_{\emptyset })N\\ \;=\left(1-{\left(1-\frac{1}{N}\right)}^{\frac{KN}{MT}}\right)N. \end{array}$$

For the weak scaling shown in Figure 1 we express *N* = *N*_VP_*MT* in terms of the number of local neurons per virtual process *N*_VP_. Using the definition of *K*_VP_, Equation (6) becomes

(7) $${\tilde{N}}_{c}^{>0}=\left(1-e^{-\frac{K_{\text{VP}}}{N}}\right)N.$$

In weak scaling the total number of local synapses *K*_VP_ remains constant and we find the limit of *N*^>0^_c_ by approximating the exponential to linear order

$$\underset{N\to \infty }{\lim}N_{\text{c}}^{>0}=\left(1-\left(1-\frac{K_{\text{VP}}}{N}\right)\right)N=K_{\text{VP}}.$$

Using Equation (7) the number of neurons *N*_ζ_ at which a fraction ζ of the maximal number of target lists *K*_VP_ contains at least one synapse is given by the relation

(8) $$\zeta K_{\text{VP}}=\left(1-e^{-\frac{K_{\text{VP}}}{N_{\zeta }}}\right)N_{\zeta }.$$

With the substitution *s* = −*K*_VP_/*N*_ζ_ the relation is of the form *e^s^* = 1 + ζ *s* and can be inverted using the Lambert-*W* function (Corless et al., 1996) yielding

(9) $$N_{\alpha }=K_{\text{VP}}\zeta {\left[1+\zeta W\left(-\frac{e^{-\frac{1}{\zeta }}}{\zeta }\right)\right]}^{-1}.$$

Starting again from Equation (8) with a second order approximation for the exponential

$$\zeta K_{\text{VP}}≃\left(1-\left(1-\frac{K_{\text{VP}}}{N_{\zeta }}+\frac{K_{\text{VP}}^{2}}{2N_{\zeta }^{2}}\right)\right)N_{\zeta }$$

the relation depends linearly on *N*_ζ_, so

(10) $$N_{\zeta }≃\frac{K_{\text{VP}}}{2\left(1-\zeta \right)}.$$

Following Equation (4) the probability of a particular neuron to establish exactly one synapse with a local neuron is

(11) $$\begin{array}{l} p_{1}={\left(1-\frac{1}{N}\right)}^{KN/MT-1}\left(\frac{1}{N}\right)\frac{KN}{MT}\\ \;≃e^{-\frac{K}{MT}}\frac{K}{MT}\\ \;≃\left(1-\frac{K}{MT}\right)\frac{K}{MT}+O\left[{\left(\frac{K}{MT}\right)}^{3}\right]. \end{array}$$

Therefore the expected number of target lists with exactly one synapse is

(12) $$\begin{array}{l} N_{\text{c}}^{1}=p_{1}N={\left(1-\frac{1}{N}\right)}^{K_{\text{VP}}-1}K_{\text{VP}}\\ \;={\left(1-\frac{1}{MTN_{\text{VP}}}\right)}^{\frac{K}{MT}-1}\frac{K}{MT}. \end{array}$$

Naturally *N*^1^_c_ has the same limit *K*_VP_ as *N*^>0^_c_. The probability to establish more than one synapse with a local neuron is the remainder

(13) $$\begin{array}{l} p_{>1}=1-p_{\emptyset }-p_{1}\\ \;=\left(1-{\left(1-\frac{1}{N}\right)}^{K_{\text{VP}}}-{\left(1-\frac{1}{N}\right)}^{K_{\text{VP}}-1}\left(\frac{1}{N}\right)K_{\text{VP}}\right)\\ \;≃1-e^{-\frac{K}{MT}}-e^{-\frac{K}{MT}}\frac{K}{MT}\\ \;≃\frac{K}{MT}-\frac{1}{2}{\left(\frac{K}{MT}\right)}^{2}-\left(1-\frac{K}{MT}\right)\frac{K}{MT}+O\left[{\left(\frac{K}{MT}\right)}^{3}\right]\\ \;=\frac{1}{2}{\left(\frac{K}{MT}\right)}^{2}+O\left({\left(\frac{K}{MT}\right)}^{3}\right), \end{array}$$

where from the second to the third line we ignored the −1 in the exponent, identified the exponential function in the limit, and from the third to the fourth line approximated the expression consistently up to second order in $\frac{K}{MT}$. The expected number of such target lists is

$$\begin{array}{l} N^{\;>1}=\left(1-{\left(1-\frac{1}{N}\right)}^{K_{\text{VP}}}-{\left(1-\frac{1}{N}\right)}^{K_{\text{VP}}-1}\left(\frac{1}{N}\right)K_{\text{VP}}\right)N\\ \;≃\frac{1}{2}K_{\text{VP}}^{2}\frac{1}{N}, \end{array}$$

which can be expressed in terms of *MT* and *N*_V*P*_ by noting that *N* = *N*_VP_*MT* and *K*_VP_ = $\frac{KN}{MT}$ = *N*_VP_*K*. The limit exposes that the number of target lists with more than one synapse declines hyperbolically with *N*.

### 2.5. Supercomputers

The compute nodes in contemporary supercomputers contain multi-core processors; the trend toward ever greater numbers of cores is further manifested in the BlueGene/Q architecture with 16 cores per node, each capable of running 4 hardware threads. These architectures feature a multi-level parallel programming model, each level potentially operating at different granularity. The coarsest level is provided by the process based distribution, using MPI for inter-process communication message passing interface (Message Passing Interface Forum, 1994). Within each process, the next finer level is covered by threads, which can be forked and joined in a flexible manner with OpenMP enabled compilers (Board, 2008). The finest level is provided by streaming instructions that make use of concurrently operating floating point units within each core.

To evaluate the scalability of NEST in terms of run time and memory usage we performed benchmarks on two different distributed-memory supercomputing systems: the JUQUEEN BlueGene/Q at the Jülich Research Centre in Germany and the K computer at the Advanced Institute for Computational Science in Kobe, Japan. The K computer consists of 88,128 compute nodes, each with an 8-core SPARC64 VIIIfx processor, which operates at a clock frequency of 2 GHz (Yonezawa et al., 2011), whereas the JUQUEEN supercomputer comprises 28,672 nodes, each with a 16-core IBM PowerPC A2 processor, which runs at 1.6 GHz. Both systems support a hybrid simulation scheme: distributed-memory parallel computing with MPI and multithreading on the processor level. In addition, the individual cores of a JUQUEEN processor support simultaneous multithreading with up to 4 threads. Both supercomputers have 16 GB of random access memory (RAM) available per compute node such that in terms of total memory resources the K computer is more than three times larger than JUQUEEN. The compute nodes of the K computer are connected with the “Tofu” (*to*rus connected *fu*ll connection) interconnect network, which is a six-dimensional mesh/torus network (Ajima et al., 2009). The bandwidth per link is 5 GB/s. JUQUEEN uses a five-dimensional torus interconnect network with a bandwidth of 2 GB/s per link.

In this study all benchmarks were run with *T* = 8 OpenMP threads per compute node, which on both systems results in 2 GB of memory per thread, and hence facilitates the direct comparison of benchmarking results between the two systems. With this setup we exploited all cores per node on the K computer but only half of the cores per node on JUQUEEN. In particular we did not make use of the hardware support for multithreading the individual processor cores of JUQUEEN already provide. In total on JUQUEEN only 8 of the 64 hardware supported threads were used.

### 2.6. Maximum-filling scaling

To obtain the maximum-filling scalings shown in Figures **7–11** we followed a two step procedure. First, based on the memory-usage model, we obtain a prediction of the maximum number of neurons fitting on a given portion of the machine. We then run a series of “dry runs,” where the number of neurons is varied around the predicted value. The dry run is a feature of NEST that we originally developed to validate our model of the simulator's memory usage (Kunkel et al., 2012b). A dry run executes the same script as the actual simulation, but only uses one compute node. This feature can be enabled in NEST at run time by the simulation script. Due to the absence of the *M* − 1 other instances, the script can only be executed up to the point where the first communication takes place, namely until after the connectivity has been set up. At this point, however, the bulk of the memory has been allocated so that a good estimate of the resources can be obtained and the majority of the simulation script has been executed. In order to establish the same data structures as in the full run, the kernel needs to be given the information about the total number of processes in the actual simulation. This procedure also takes into account that of the nominal amount of working memory (e.g., 16 GB per processor on K) typically only a fraction (13.81 GB per processor on K) is actually available for the user program.

## 3. Results

### 3.1. Memory usage in the petascale regime

**Figure 2:**
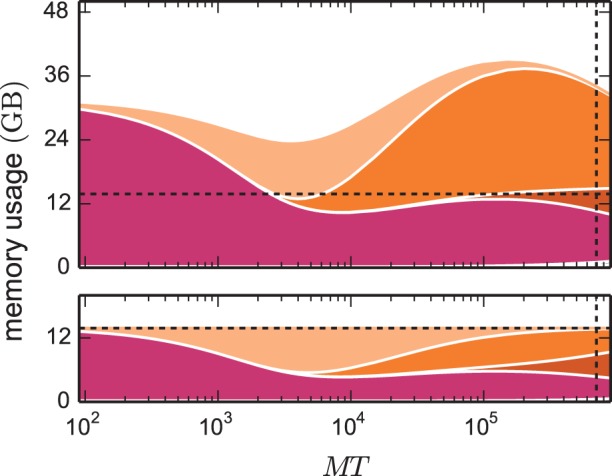
**Predicted cumulative memory usage as a function of number of virtual processes for a maximum-filling scaling**. Contributions of different data structure components to total memory usage 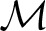 (*M*, *T*, *N*, *K*) of NEST for a network that just fits on *MT* cores of the K computer when using the 4g kernel with *T* = 8. Contributions of synapse objects and relevant components of connection infrastructure are shown in pink and shades of orange, respectively. Contributions of base memory usage, neuron objects, and neuron infrastructure are significantly smaller and hence not visible at this scale. *K* = 11,250 synapses per neuron are assumed. Dark orange: sparse table, orange: intermediate infrastructure containing exactly 1 synapse, light orange: intermediate infrastructure containing more than 1 synapse. Predicted memory usage is shown for 3g (upper panel) and 4g technology (lower panel) with identical scaling of the vertical axes. Vertical dashed black lines indicate full size of the K computer; horizontal dashed black lines indicate maximum memory usage measured on K.

The kernel of NEST 2.2 (3g kernel) is discussed in detail in Kunkel et al. (2012b) and Helias et al. (2012). In Figure 2 we compare the memory consumptions of the 3g kernel and the 4g kernel depending on the number of employed cores *MT*. We choose the number of neurons such that at each machine size the 4g kernel consumes the entire available memory. For the same network size, we estimate the memory consumption that the 3g kernel would require. The upper panel of Figure 2 shows the different contributions to memory consumption for this earlier kernel. In the following we identify the dominant contributions in the limit of large machines used to guide the development of the 4g kernel. The resulting implementation of the 4g kernel is described in Sections 3.2 to 3.5.

**Figure 3:**
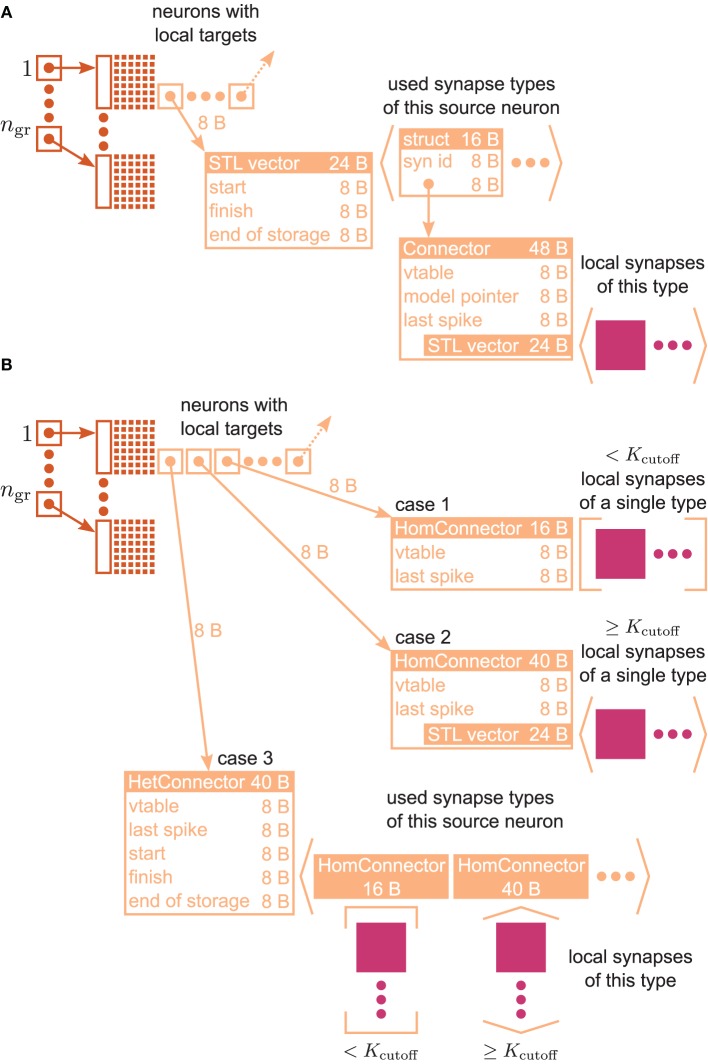
**VP-local connection infrastructure of NEST**. A sparse table (dark orange structure and attached light orange boxes with arrows) holds the thread-local connection objects (pink squares) sorted according to the global index of the associated presynaptic node. The sparse table consists of *n*_gr_ equally-sized groups, where each group maintains a bit field (tiny squares) with one bit for each global node index in the group indicating the presence or absence of local targets. If a particular node has local targets, the sparse table stores a pointer to an additional inner data structure (light orange), which has undergone a major redesign during the software development process that led from the 3g to the 4g kernel. **(A)** Connection infrastructure of the 3g kernel; listed Byte counts contribute to *m*^+^_c_ [see Equation (3)]. The inner data structure consists of a vector, which holds a struct for each connection type that the node has locally in use. Each struct links the id of a particular connection type with a pointer to the Connector that stores the connection objects of this type in a vector. **(B)** Auto-adjusting connection infrastructure of the 4g kernel. Case 1: A particular node has less than *K*_cutoff_ local connections and all are of the same type. A lightweight inner structure (HomConnector) stores the connection objects in a fixed-size array. Listed Byte counts contribute to *m*^1^_c_. Case 2: A particular node has at least *K*_cutoff_ local connections and all are of the same type. A HomConnector stores the connection objects in a dynamically-sized vector. Case 3: The local connections of a particular node are of different types. A HetConnector, which is derived from C++ vector, holds a HomConnector (either Case 1 or 2) for each connection type that the node has locally in use.

In simulations running *MT* ~ 100 (we use ~ to read “on the order of”) virtual processes, synapse objects take up most of the available memory. Hence, on small clusters a good approximation of the maximum possible network size is given by *N*_max_ ≈ 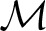
_max_/(*Km*_c_) where 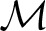
_max_ denotes the amount of memory available per MPI process. In the range of *MT* ~ 1000 virtual processes we observe a transition where due to an insufficient parallelization of data structures the connection infrastructure (shades of orange) starts to dominate the total memory consumption. For sufficiently small machine sizes *MT* <~ 1000 the intermediate synaptic infrastructure (shown in orange in Figure 3A) typically stores on each virtual process more than one outgoing synapse for each source neuron. The entailed memory overhead is therefore negligible compared with the memory consumed by the actual synapse objects. As *MT* increases, the target lists become progressively shorter; the proportion of source neurons for which the target lists only store a single connection increases. We obtain a quantitative measure by help of the memory model presented in Section 3.1, considering the limit of very large machines where *K*/(*MT*) ≪ 1. In this limit we can consistently expand all quantities up to second order in the ratio $\frac{K}{MT}$ ≪ 1. The probability (5) for a source neuron on a given machine to have an empty target list approaches unity *p*_0_ → 1. Correspondingly, the probability for a target list with exactly one entry (11) approaches *p*_1_ → $\frac{K}{MT}$. Target lists with more than one entry become quadratically unlikely in the small parameter $\frac{K}{MT}$ (13),

$$p_{\;>1}≃\frac{1}{2}{\left(\frac{K}{MT}\right)}^{2}.$$

Two observations can be made from these expressions. In the sparse limit, the probabilities become independent of the number of neurons. For small $\frac{K}{MT}$, target lists are short and, if not empty, typically contain only a single connection. To illustrate these estimates on a concrete example we assume the simulation of a network with *K* = 10^4^ synapses per neuron distributed across the processors of a supercomputer, such as the K computer, with *M* ≃ 80,000 CPUs and *T* = 8 threads each. The above estimates then yield *p*_∅_ ≃ 0.984, *p*_1_ ≃ 0.015, and *p*_>1_ ≃ 0.00012. Hence, given there is at least one connection, the conditional probability to have one synapse is $\frac{p_{1}}{p_{1}+p_{>1}}$ ≃ 1 − $\frac{1}{2}\frac{K}{MT}$ ≃ 0.992 and the conditional probability to have more than one synapse is only $\frac{p_{>1}}{p_{1}+p_{>1}}$ ≃ $\frac{1}{2}\frac{K}{MT}$ ≃ 0.008.

Figure 1 shows the number of non-empty target lists under weak scaling as a function of the network size *N*. In the limit of large networks this number approaches $\frac{NK}{MT}$, and is thus equal to the number of local synapses terminating on the respective machine. The size of the network *N*_ζ_ at which the number of target lists has reached a fraction ζ ≃ 1 of the maximum number of lists is given by *N*_ζ_ (10) with *N*_ζ_ ≃ $N_{\zeta }≃\frac{K_{\text{VP}}}{2(1-\zeta )}$. The term depends linearly on the number of synapses per virtual process. A fraction of ζ = 0.95 is thus already reached when the network size *N*_ζ_ ≃ *K*_VP_/(2·0.05) = 10 *K*_VP_ exceeds the number of local synapses by one order of magnitude independent of the other parameters. This result is independent of the detailed parameters of the memory model, as it results from the generic combinatorics of the problem.

The effects on the memory requirements can be observed in Figure 2 (top), where the amount of memory consumed by lists of length one and larger one are shown separately: at *MT* ~ 10^4^ the memory consumed by the former starts exceeding the consumption of the latter. At *MT* ~ 10^5^ the memory consumed by lists with more than one synapse is negligible. As we have seen in Section 2.5, the scenario at *MT* ~ 10^5^ is the relevant one for currently available supercomputers. In the following we use the analysis above to guide our development of memory-efficient data structures on such machines. Figure 2 (top) highlights that the intermediate synaptic infrastructure when storing only a single synapse must be lean. In the 3g kernel the memory consumed by a single synapse object is 48 B, while the overhead of the intermediate infrastructure is 136 B. Hence in the limit of sparseness, a synapse effectively costs 48 B + 136 B. Reducing the contribution of the intermediate infrastructure is therefore the first target of our optimizations described in Section 3.2. We identify the size of the synapse objects as the contribution of secondary importance and describe in Section 3.3 the corresponding optimization. The resulting small object sizes can only be exploited with a dedicated pool allocator (see Section 3.4). The least contribution to the memory footprint stems from the neuronal infrastructure, the improved design of which is documented in Section 3.5. The sparse table has an even larger contribution than the neuronal infrastructure. However, the employed collective communication scheme that transmits the occurrence of an action potential to all other machines requires the information whether or not the sending neuron has a target on a particular machine. This information is represented close to optimal by the sparse table. In the current work we therefore refrained from changing this fundamental design decision.

### 3.2. Auto-adjusting connection infrastructure

Figure 3A illustrates the connection infrastructure of NEST in the 3rd generation kernel (3g). As shown in Section 3.1 this data structure produces an overhead in the limit of virtual processes *MT* exceeding the number of outgoing synapses *K* per neuron; a presynaptic neuron then in most cases establishes zero or one synapse on a given core. The overhead can be avoided, because the intermediate data structure is merely required to distinguish different types of synapses originating from the same source neuron. For only a single outgoing synapse per source neuron it is not required to provide room to simultaneously store different types.

**Figure 4:**
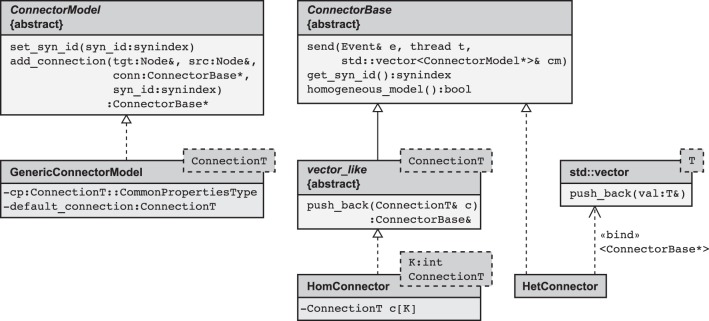
**Simplified class diagram of the connection infrastructure**. The base class ConnectorModel serves as a factory for connections. The member function add_connection is called when a new synapse is created and implements the algorithm to select the appropriate storage container (see also Algorithm 2). The derived template GenericConnectorModel contains all code independent of synapse type and is instantiated once for each synapse type. It also holds the set of default values for synapse parameters (data member default_connection) and those parameters that are the same for all synapses of the respective type (data member cp). The class ConnectorBase defines the interface for all synapse containers, providing abstract members to deliver spike events (send), to determine the synapse type of type-homogeneous containers (get_syn_id), and to test if a container is type-homogeneous, i.e., whether it stores a single or several different synapse types (homogeneous_model). Two derived classes implement type-homogeneous containers (HomConnector) and type-heterogeneous containers (HetConnector). The latter, in turn, may contain several containers of class HomConnector, stored in a C++ standard library vector (by double inheritance from std::vector). HomConnector inherits the function push_back from the interface vector_like, required to implement the recursive sequence of container types, described in Algorithm 1.

The main idea is hence to use data structures that automatically adapt to the stored information, as illustrated in Figure 3B. The algorithm wiring the network then chooses from a set of pre-defined containers depending on the actual need. The corresponding data types are arranged in a class hierarchy shown in Figure 4, with the abstract base class ConnectorBase defining a common interface. The wiring algorithm distinguishes four cases, depending on the number and types of the outgoing synapses of the given source neuron:

**Case 0** The source neuron has no target on this machine. In this case, the unset bit in the sparse table (see Figure 3) indicates the absence of synapses and no further data structures are created.
**Case 1** The source neuron has outgoing synapses that are of the same type. In this case we use a type-homogeneous container HomConnector. Depending on the number of synapses, we use two different strategies to implement the homogeneous container. If less than *K*_cutoff_ synapses are stored, we employ a recursive C++ template definition of a structure that holds exactly 1, 2, …, *K*_cutoff_ synapses. Here *K*_cutoff_ is a compile-time constant that throughout this work was chosen to be *K*_cutoff_ = 3. The recursive template definition is shown in Algorithm 1 and follows the known pattern defining the recursion step with an integer-dependent template and the recursion termination by a specialization for one specific integer value (Vandervoorde and Josuttis, 2003, Ch. 17). The classes are instantiated at compile time due to the recursive definition of the method push_back. The set of containers implements the functionality of a vector, requiring just the memory for the actual payload plus an overhead of 8 B for the virtual function table pointer due to the use of an abstract base class providing the interface of virtual functions. Our implementation uses a custom-made pool allocator ensuring that each thread allocates from its own contiguous block of memory to improve the cache performance and to reduce the overhead of allocating many small objects (see Section 3.4).
**Case 2** If more than *K*_cutoff_ synapses of the same type are stored we resort to the conventional implementation employing a std::vector from the C++ standard template library. The implementation of a vector entails an additional overhead of 3 times 8 B. This case provides the recursion termination for the set of homogeneous containers, as shown in Algorithm 1.
**Case 3** If a source neuron has synapses of different types targeting neurons on the same machine, we employ the container HetConnector. This intermediate container stores several homogeneous connectors (of either Case 1 or 2 above) and is inherited from a std::vector<ConnectorBase^*^>.

The algorithm for creating new connections employing these adaptive data containers is documented as pseudo-code in Algorithm 2.

### 3.3. Condensed synapse objects

In a typical cortical neuronal network model, the number of synapses exceeds the number of neurons by a factor ~ 10^4^. To reduce the memory consumption of a simulation, it is thus most efficient to optimize the size of synapse objects, outlined in our agenda at the end of Section 2.3 as step two of our optimizations. We therefore reviewed the Connection class (representing the synapses) and identified data members that could be represented more compactly or which could even be removed. In refactoring the synapse objects our objective is to neither compromise on functionality nor on the precision of synaptic state variables, such as the synaptic weight or the spike trace variables needed for spike-timing dependent plasticity (Morrison et al., 2007); these state variables are still of type double in the new simulation kernel (4g). Our analysis of the Connection data structure identified three steps to further reduce the memory consumption of single synapse objects. The steps are explained in the following three subsections and concluded by a subsection summarizing the resulting reduced memory footprint.

#### 3.3.1. Avoidance of polymorphic synapse objects

As shown in Figure 4 and in Algorithm 1, the container-classes are templates with the synapse type connectionT as a template parameter. Consequently, the container itself does not need a polymorphic interface, because by specialization for a particular synapse type this type is known and fixed at compile time. The only exception to this rule is synapse creation: we here need to check that the synapse model and the involved neuron models are compatible. More precisely, we need to ensure that (i) the new connection and (ii) the target node are able to handle the type of events sent by the source node. In NEST 2.2 (3g) the first of the two checks (i) requires a common interface to all synapse objects (i.e., an abstract base class Connection) that provides a set of virtual functions, one for each possible event type (spike events, voltage measurement requests, etc.). The synapse object then implements only those virtual member functions for event types it can handle. A similar pattern is used for the check (ii), determining whether the target node is able to handle the incoming event type. On a 64 bit architecture the virtual base class causes a per-object overhead of 8 B for the virtual function table pointer. Having such a pointer in each node hardly affects the memory consumption, but spending these additional 8 B per synapse object seems rather costly given the fact that the connection handshake is the only instance when NEST exploits the polymorphism of Connection. Therefore, in the 4g kernel we redesigned the handshaking algorithm such that it still makes use of the polymorphism of Node but no longer requires virtual functions in Connection. This reduces the per-synapse memory usage *m*_c_ by 8 B.

**Figure 5:**
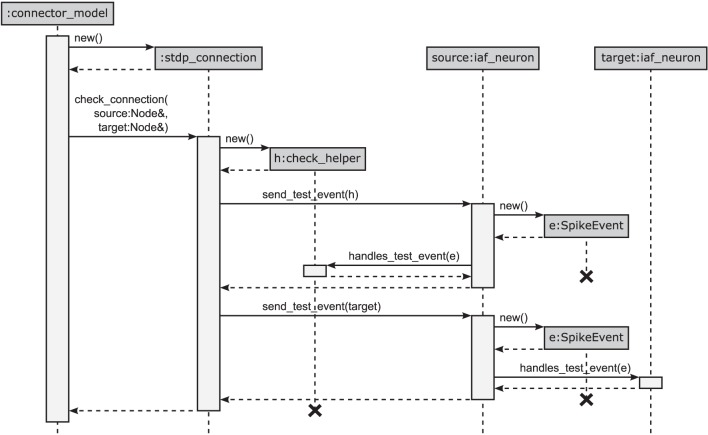
**Connection handshaking mechanism of the 4g kernel**. By executing connect, NEST calls add_connection of the corresponding connector in the connection infrastructure. This function creates a new instance for the connection, sets its parameters and starts the connection handshaking by calling check_connection. This function creates an instance of the check_helper class and calls send_test_event of the source node twice to send test events to the synapse represented by check_helper and to the target node, respectively. Both instances execute handles_test_event which, if the event cannot be handled, ends in the base-class implementation throwing an exception.

The design pattern that circumvents polymorphic synapse objects is derived from the visitor pattern (Gamma et al., 1994; Alexandrescu, 2001). A sequence diagram of the connection setup is shown in Figure 5. The crucial step is to shift the set of virtual functions that check the validity of received events from the synapse objects to a nested class, called check_helper. Each connection class owns its specific version of check_helper, which is derived from the Node base class. This inner class redefines the virtual function handles_test_event for those event types the connection model can handle. The default implementations inherited from the base class throw an exception and thus by default signal the inability of the synapse to handle the particular event. Since the connection class only contains the nested class definition, rather than a member of type check_helper, the nested class does not contribute to the memory footprint of the connection.

This new design has the additional advantage that checks (i) and (ii) have the same structure following the visitor pattern, shown in Figure 5: For check (i), the synapse creates an object of its corresponding type check_helper, passes it as a visitor to the source neuron's member function send_test_event, which in turn calls the overloaded version of the virtual function handles_test_event that has the matching event type. The test passes if handles_test_event is implemented for the type of event e, shown for the example of a SpikeEvent in Figure 5. The second check (ii) proceeds along analogous lines. Here the target neuron is passed as a visitor to the source neuron's member function send_test_event, which in turn sends the event via a call to the target neuron's handles_test_event method.

#### 3.3.2. Indexed target addressing

A connection has to store the information about its target node. In NEST 2.2 (3g) this was solved by storing a pointer to the target node, consuming 8 B on a 64 bit architecture. The new simulation kernel (4g) implements the target addressing flexibly via a template argument targetidentifierT to the connection base class. This template parameter enables the formulation of synapse objects independent of how the target is stored, as long as the targetidentifierT provides methods to obtain the actual target neuron's physical address via a member get_target_ptr(). Besides the original implementation that stores the target as a full pointer, the 4g kernel supports indexed addressing of the target neuron. The two addressing schemes correspond to different implementations of the target identifier. Each synapse type may be instantiated with either of them.

Indexed addressing makes use of the limited number of neurons that are local to a given virtual process. These nodes are stored in a vector of pointers required to update the neuronal dynamics. In a parallel simulation, the number of local neurons rarely exceeds ~ 10^4^ nodes. The index space of local nodes is thus sufficiently covered by indices of 2 B length corresponding to a maximal index of 2^16^ − 1 = 65,535. The target identifier then stores the index of the target neuron corresponding to its position in the vector of VP-local nodes. We call this index “thread-local id” in the following. Determining the target requires one more indirection to compute the actual address from the stored index, but saves 6 B of memory per target pointer. The implementation requires an extension of the user interface, because in the 3g kernel the thread-local vectors of nodes are only generated dynamically at the start of the simulation. The translation from the global id (GID) of a target neuron to the thread-local id is hence unavailable during wiring of the network. To employ the indexed addressing scheme the user therefore needs to execute the function CreateThreadLocalIds after all nodes have been created and prior to the creation of any synapse using the indexed addressing scheme. This function fills the vectors holding the thread-local neurons and moreover stores the thread-local id for each neuron in its base class. The latter information is needed during connection setup to obtain the thread-local id from a given global neuron id.

#### 3.3.3. Combined storage of delay and synapse type

In a third step, the storage of the synapse type (syn_id) and the delay (d) of a connection are optimized. The 3rd generation connections stored these properties separately, the delay as a long integer variable with 8 B and the synapse type as an unsigned integer with 4 B length. The delay is represented as an integer multiple of the chosen simulation resolution *h*, typically *h* = 0.1 ms (Morrison et al., 2005b). A reduction of the storage size from 8 B to 3 B hence corresponds to a restriction of the maximum delay (assuming *h* = 0.1 ms) from 2^64^ *h* ≃ 5.8 · 10^7^ years to 2^24^ *h* ≃ 1670 s, which is more than sufficient for all practical demands. The limitation of the synapse type identifier to an unsigned int of 1 B limits the number of different synapse types to 2^8^ = 256, a reasonable upper limit for different synapse models. To ensure that delay and synapse identifier are stored as compactly as possible independent of memory alignment choices of the compiler, both variables are stored as 8 bit and 24 bit fields (Stroustrup, 1997, Ch. C.8.1) in a common structure requiring 4 B; the structure provides methods for convenient access to synapse id and delay. Overall, the new storage scheme thus requires just 4 B per synapse instead of 8 B + 4 B required by the 3rd generation kernel for the same information, saving 8 B of memory.

#### 3.3.4. Memory footprint of synapse objects

The simplest type of synapse, the static synapse, is described completely by information identifying its target, type, delay and weight. In the 3g simulation kernel, the type required 4 B, while the three other items each required 8 B as described above, for a total of 28 B. On computers with 64 bit architecture, data types requiring 8 B, such as pointers, doubles and long integers, are by default aligned with 64 bit-word boundaries, so that a static synapse object in effect required 32 B: the 4 B unsigned integer representing the synapse type was padded to 8 B to ensure proper alignment of the following 8 B variable.

Combining all three 4g kernel optimizations described above, the size of a static synapse object shrinks to 2 B for the target index, 4 B for the combined synapse type id and delay, and 8 B for the synaptic weight (double precision). Provided that the data members in the synapse object are ordered as is {target index, synapse type id + delay, synaptic weight}, the object requires 16 B in total, including 2 B of padding inserted after the target index.

In addition to the member variables of a static synapse, an object representing an STDP synapse stores the synaptic trace variable (double) (Morrison et al., 2008), consuming another 8 B. The total memory footprint for STDP synapse objects is thus 24 B, of which 22 B or 92% hold data, while the remaining 8% are padding. On some systems, the 2 B of padding might in principle be avoided by forcing the compiler to generate code for unaligned memory access, e.g., using the __attribute__(packed) provided by the GNU C++ compiler (Free Software Foundation, 2013, Ch. 6.36). Unfortunately, such packing is not defined in the C++-standard and thus compiler-dependent; on some architectures, unaligned memory access may also incur significant runtime overhead (Rentzsch, 2005). Given that synapse objects contribute less than 50% to the total memory requirement of the simulator for large networks, as shown in Figure 2, the memory overhead incurred by padding is thus less than 4%. To ensure maximum portability, NEST does not use unaligned memory access.

### 3.4. Pool allocator

The standard allocator on most systems has two severe problems with regard to memory locality when using multiple threads and memory overhead when allocating many small objects. The first problem stems from the allocator not taking into account the actual physical layout of the memory architecture of the machine. In particular, the memory for objects of different threads is often not separated, but objects are rather allocated in the order in which they are created. This makes caching on multi-core machines with different caches for different cores or with different memory banks for different cores (cf. ccNUMA) inefficient and leads to frequent (and slow) cache reloads from the main memory, a problem generally referred to as cache thrashing. The second problem is caused by the fact that the allocator needs to keep administrative information about each single allocation for freeing the memory and returning it to the operation system after its use. Usually, this information consists at least of a pointer to the data (8 B) and the size of the allocated memory (8 B). If the size of administrative data is in the range of the size of the allocation, this means a significant memory overhead.

To ameliorate these problems, we implemented a stateless custom allocator, which does not keep any administrative information about allocations and provides thread-local memory pools for the storage of connection containers (see Stroustrup, 1997, Ch. 19.4.2 for the basic concept). The absence of allocation information is not a problem, as the data structures for the storage of synapses only grow, and never are freed during the run of the program. Moreover, as shown in Section 3.1, in the sparse limit reallocation of target lists is rare, as most target lists contain only a single synapse. In this limit the simple pool allocator is hence close to optimal. The use of the pool allocator needs to be selected by the user with a compile-time switch. On small clusters or desktop machines, where frequent reallocation takes place, the standard allocator is recommended.

### 3.5. Sparse node array

Kunkel et al. (2012b) showed that the memory overhead required to represent neurons on each MPI process could be reduced considerably by using a sparse table. In such a table, each non-local neuron is represented by a single bit, while the overhead for local neurons is only a few Bytes. Kunkel et al. (2012b) give the following expression for the memory required to represent neurons:

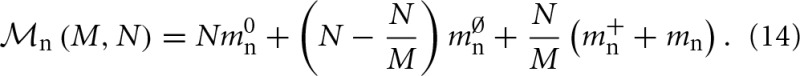



Using the sparse table, one has the following parameters: *m*^0^_n_ = $\frac{1}{3}$ B, *m*^∅^_n_ = 0 B, *m*^+^_n_ = 24 B, and *m*_n_ ≈ 1000 B, where the first three parameters describe overhead, while the last parameter represents actual neuron objects. With *N*/*M* ~ 10^3^ neurons per MPI process, neuron objects consume ~ 1 MB, while the overhead from Equation (14) is

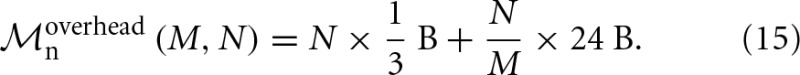



For *N*/*M* ~ 10^3^, the second term is about 24 kB and thus negligible, while the first term becomes appreciable for large networks. Indeed, for *N* = 10^8^ neurons, it amounts to approximately 32 MB, for *N* = 10^9^ to 318 MB, almost all of which are consumed for zero-bits in the bit array of the sparse table. This not only requires appreciable amounts of memory to represent vanishing amounts of information, it also means that sparse table lookups, which in most cases return negative results, become cache inefficient, as the size of the bit array by far exceeds the cache size.

In the following we show how this overhead can be eliminated entirely. The basic idea is to exploit the round-robin distribution of neurons to virtual processes and thus MPI processes (Eppler, 2006; Plesser et al., 2007). As NEST builds a network, neurons are created with strictly increasing global neuron ids (GIDs) *g*, and each neuron is assigned to and stored on MPI rank

(16) $$m_{g}=g\;\mathrm{mod}\;M\;.$$

Thus, if we place the neurons assigned to a single MPI process in an array in order of creation, GIDs of neighboring neurons will differ by *M*. For any given *g*, we thus can use linear interpolation between the GIDs of the first and last local neuron to determine the index pertaining to that GID in the array of local neurons. We then only need to check whether the neuron found at that index has the correct GID (i.e., is local to the MPI process) or not, in the latter case we conclude that the neuron is managed by a different MPI process.

Unfortunately, reality is slightly more complicated. Certain network elements are replicated on all virtual processes in NEST, either of logical necessity (subnet nodes used to structure large networks) or for performance reasons (stimulating and recording devices); we refer to such network elements as replicated nodes. This means that (i) any node *g* is guaranteed to be local to MPI rank *m*_*g*_ according to Equation (16), (ii) if node *g* is a replicating node, it is also local to all other MPI ranks, and thus (iii) neighbors in the array of nodes are not necessarily spaced in intervals of *M*. This will skew the linear interpolation used to look up nodes by their GIDs.

Fortunately, the number of replicating nodes must be small in any practical simulation, because the memory requirement of the simulation would not scale otherwise. Thus, the skew will be small. This suggests the following approach: Let *n*_loc_ be the number of local nodes for a given MPI rank and *g*_loc,min_ and *g*_loc,max_ the smallest and largest local GID, respectively, (we ignore that the root network with GID 0 is local to all ranks), and define the scaling factor

(17) $$\alpha =\frac{n_{\text{loc}}-2}{g_{\text{loc},\text{ max}}-g_{\text{loc},\text{ min}}}\;.$$

Then

(18) $$l_{g}*=\left\lfloor 1+\alpha (g-g_{\text{loc},\text{ min}})\right\rfloor$$

provides a linear estimate of the index of the node with GID *g* in the local array of nodes. This estimate is exact if we have a single MPI process or there are no replicating nodes except the root network. We can thus look up nodes by starting our search at index l^*^_g_ in the array of local nodes and then proceed to smaller or larger nodes until we either have found the node with GID *g* or reached a node with GID smaller (larger) than *g*, in which case we conclude that node *g* is not local. The complete algorithm is given as Algorithm 3.

The underlying data structure is the SparseNodeArray: Its main component is a C++ vector that stores in each element the pointer to a node and the GID of that node. This ensures that the linear search from l^*^_g_ is cache efficient, compared to a vector keeping node pointers only, which would require looking up the GID via the node pointer in each search step. Additionally, the SparseNodeArray keeps track of the largest GID in the entire network (to detect invalid GIDs), the smallest and largest local GID and the scaling factor α. The memory overhead for this data structure is thus one pointer and one long per *local* node, i.e.,

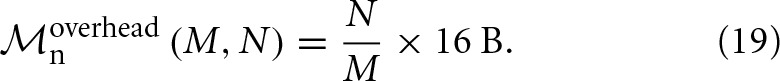



The overhead now only depends on the number of neurons per process, *N*/*M*, but no longer directly on the total number of neurons in the network, *N*.

To evaluate the quality of the linear estimate provided by Equation (18), we counted the number of search steps required to find the correct node using a specially instrumented version of the NEST code. We collected data for *M* = 2048 to *M* = 65,536 MPI processes with four threads each and between 550 and 230 nodes per thread. The average number of search steps is 0.7 in this data and lookup never requires more than two steps for over 3 billion analyzed lookups. Therefore, we consider the linear estimate to be close to optimal. In addition to the lookup by GID, the SparseNodeArray also provides an interface for direct iteration solely over the local nodes to allow for, e.g., efficient initialization of all nodes.

### 3.6. Performance of 4g technology

**Figure 6:**
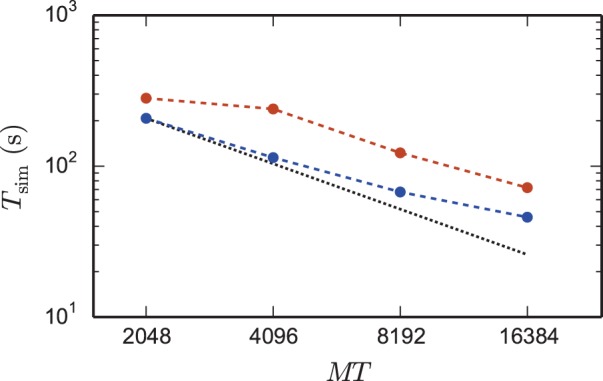
**Strong scaling on K computer and JUQUEEN**. Solid curves show the scaling of simulation time, dashed curves show setup time for networks of *N* = 5,242,880 neurons on JUQUEEN (blue) and *N* = 5,210,112 on the K computer (red). Black dotted lines are the linear expectations for simulation and setup. All simulations were carried out using the parameters of set 1 (cf. Section 2.2).

Figure 6 shows a strong scaling for the 4g kernel. At the left-most point the workload per core is maximal; the number of neurons is chosen such that the memory is completely filled. Near the point of maximum workload the scaling on JUQUEEN is close to optimal, but degrades for lower workload. This is expected as ultimately the serial overhead dominates the simulation time. The relevance of a strong scaling graph is therefore naturally limited. To minimize the queuing time and the energy consumption it is desirable to choose the smallest possible machine size that enables the simulation of a given problem size. In the following we will hence study maximum-filling scalings, where for a given machine size *MT* we simulate the largest possible network that completely fills the memory of the machine. The procedure to arrive at the maximum possible network size is described in Section 2.6.

**Figure 7:**
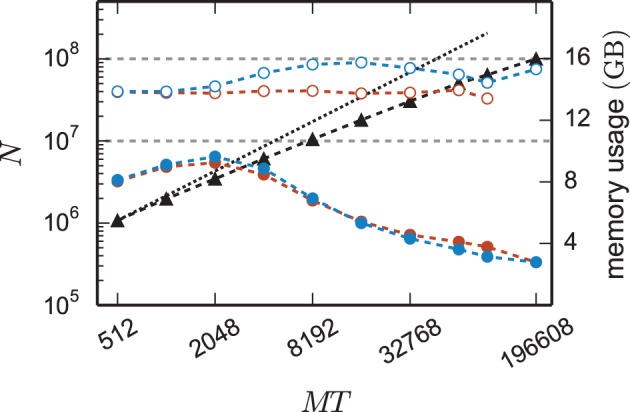
**Comparison of memory usage of 3g and 4g technology**. Black triangles show the maximum possible network size, using parameters of set 1 (cf. Section 2.2), that can be simulated on the K computer and on JUQUEEN when using the 3g technology with *T* = 8 threads per compute node (left vertical axis). The dotted black line indicates the ideal case where the network size increases with the same factor as the number of virtual processes. Circles show the memory consumption (right vertical axis) of simulations with the 3g (open) and 4g (filled) technology on the K computer (red) and JUQUEEN (blue), respectively.

The reduction of the memory consumption of the 4g kernel compared to the 3g kernel is shown in Figure 7. At a given machine size *MT* the same network is simulated with both kernels. For each *MT* the size of the network is chosen such that the 3g simulation consumes all available memory on the K computer (maximum-filling scaling). At high numbers of cores around *MT* ~ 100,000 the 4g kernel reduces the required memory by a factor of more than 3, for smaller machine sizes the reduction of memory consumption is less, but still substantial.

**Figure 8:**
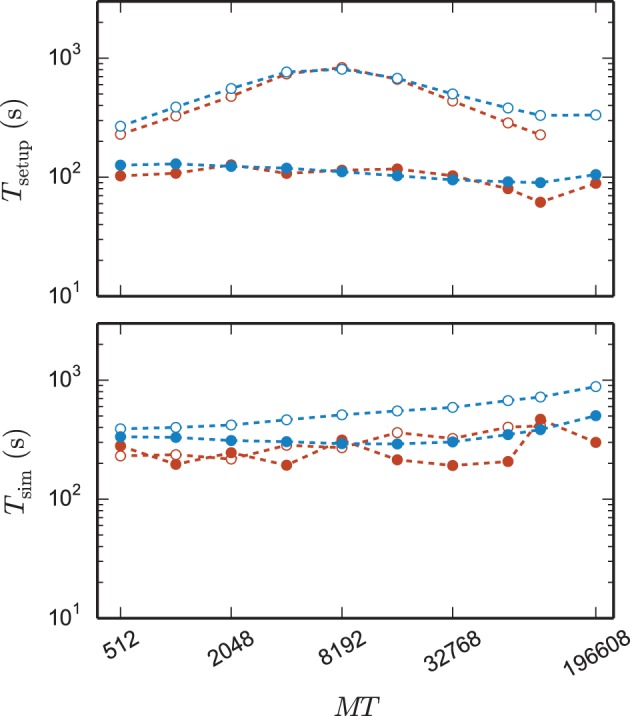
**Comparison of performance of 3g and 4g technology**. Setup time (upper panel) and simulation time (lower panel) as a function of number of virtual processes for the maximum network size that can be simulated when using the 3g technology (see Figure 7) and parameter set 1 (cf. Section 2.2). Circles refer to simulations with 3g (open) and 4g (filled) technology, respectively. Results are shown for the K computer (red) and JUQUEEN (blue) using *T* = 8 threads per compute node.

Figure 8 shows the time to setup the network (top panel) and the simulation time (bottom panel) for the 3g and the 4g kernel in the same maximum-filling scaling as in Figure 7. Although both kernels simulated the same network at a given *MT* and hence the same computation takes place in both simulations (same update steps of neurons and synapses, same activity, etc.), the run time of the new kernel is typically reduced especially at large machine sizes. On JUQUEEN this reduction monotonically increases with machine size, on the K computer the simulation time exhibits fluctuations that presumably originate from different load levels caused by other users of the machine, potentially occluding a clear monotonic dependence. The faster simulation time of the new kernel points at the random memory access as an important contribution to the computation time. The smaller objects of the connection infrastructure enable more efficient use of the cache and reduce the overall required memory bandwidth.

The 4g implementation exhibits a reduction in setup time by a factor of 2–8 depending on network size (Figure 8, top panel). In parts this higher performance is due to ongoing conventional optimization of the wiring routines; for example, by representing consecutive neuron ids within the wiring process by the beginning and end of the range (4g) instead of by explicitly naming all elements (3g). In parts the difference is due to faster memory allocation through the dedicated pool allocator and the smaller objects representing synapses and connection infrastructure.

**Figure 9:**
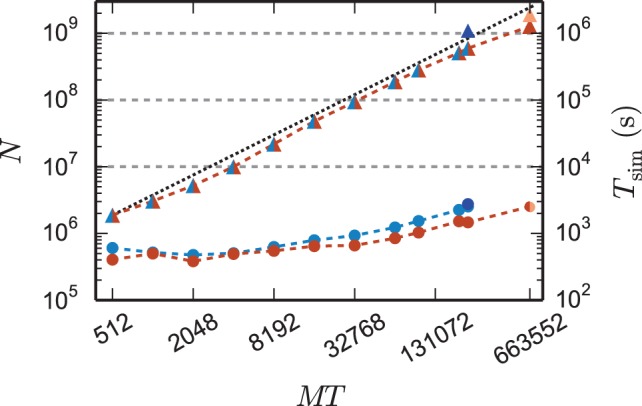
**Maximum network size and corresponding run time as a function of number of virtual processes**. Triangles show the maximum network size that can be simulated with parameter set 1 (cf. Section 2.2) on the K computer (red) and on JUQUEEN (blue) when using the 4g technology with *T* = 8 threads per compute node. The dotted black line indicates the ideal case where the network size increases with the same factor as the number of virtual processes. Dark blue (JUQUEEN) and orange (K) triangles represent the maximum network size using all compute nodes and parameter set 2 (JUQUEEN: 1.08 · 10^9^ neurons, K: 1.86 · 10^9^ neurons). Filled circles show the corresponding wall-clock time required to simulate the network for 1 s of biological time.

The comparison of the 3g and 4g kernels in Figures 7, 8 is based on the maximum network size the 3g kernel can represent on a given number of cores. The reduced memory consumption of the 4g kernel allows us to simulate larger networks with the same computational resources. In Figure 9 we therefore show a maximum-filling scaling determined for the 4g kernel, showing the maximum network size that can be simulated with a given number of cores *MT* and hence for a given amount of working memory. The growth of network size *N* with *MT* stays close to the ideal line.

Using parameter set 2 with *K* = 6000 synapses per neuron and employing all 82,944 nodes of the K computer simultaneously in a single simulation with 8 cores each, we reached a maximum network size of 1.86 · 10^9^ neurons and a total of 11.1 · 10^12^ synapses. This is the largest simulation of a spiking neuronal network reported so far (RIKEN BSI, 2013). This world-record simulation was performed on K, because only this machine provides the necessary memory to represent all synapses of the simulation with generic connectivity. Access to JUQUEEN and its predecessor JUGENE was, however, crucial for the design and implementation of the simulation kernel during the development phase of the K computer and for performing smaller simulations testing the implementation (Diesmann, 2012). In terms of memory, the K computer is at the time of writing the second largest computer (1.4 PB RAM, on the nodes available here 1.3 PB), exceeded only by the IBM sequoia computer (1.5 PB) at the Lawrence Livermore National Laboratory. JUQUEEN provides about one-third (0.46 PB) of the memory of the former two systems. Previous to the current report, the largest spiking network simulation comprised 1.62 · 10^9^ neurons with on average about 5700 synapses per neuron (Ananthanarayanan et al., 2009). The simulation required less than 0.144 PB of memory by making use of a specific modular connectivity structure of the network. Thus, in contrast to the case of an arbitrary network discussed in the present study, the choice of a specific structure enabled the authors to condense the memory components of the connectivity infrastructure, corresponding in our implementation to the sparse table, the intermediate infrastructure, and the synapses (see Figure 2), into effectively only 16 B per synapse.

Comparing the theoretical prediction of the memory model to the empirically found maximum network size reveals that the theory underestimates the actual memory consumption. As shown in our previous work (Kunkel et al., 2012b) these deviations are most likely due to underestimation of the object sizes. A direct comparison of the memory consumption of *n* objects of size *m* to the predicted value *nm* typically uncovers non-optimal object alignment. Obtaining instead the effective object sizes by linear regression, as in our earlier work (Kunkel et al., 2012b), would decrease the deviation of the model from the empirical result.

**Figure 10:**
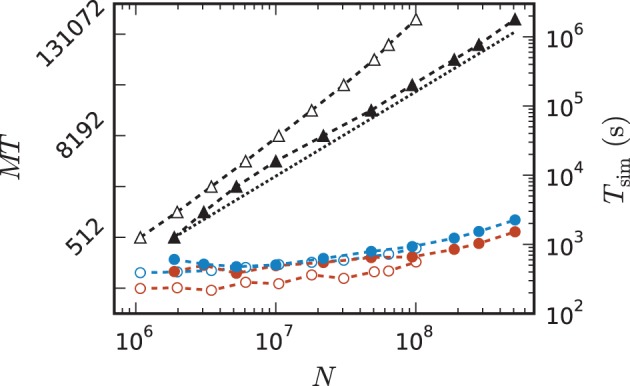
**Comparison of maximum-filling scaling for 3g and 4g**. Required number of cores *MT* (black, left vertical axis; numbers quadrupling between ticks) and time (colors, right vertical axis) as a function of network size for a maximum filling with parameter set 1 (cf. Section 2.2) for the 3g kernel (open symbols) and the 4g kernel (filled symbols). Benchmarks on the K computer shown in red, on JUQUEEN in blue. The dashed black line indicates the ideal case where the required number of cores increases only with the same factor as the network size.

Comparing the memory resources required by the 3g and 4g kernels as a function of the just fitting network size (Figure 10) shows the new kernel to be closer to the optimal linear scaling: doubling the machine size nearly doubles the network size that can be simulated. At *MT* = 196,608 virtual processes, the maximum network size possible with the 4g kernel is *N*^4g^_max_ = 5.1 · 10^8^ compared to *N*^3g^_max_ = 1.0 · 10^8^ for the 3g kernel. The absolute simulation time increases in the maximum-filling scheme for the 4g kernel compared to the 3g kernel, because the higher number of neurons per core constitutes a proportionally larger workload per core.

**Figure 11:**
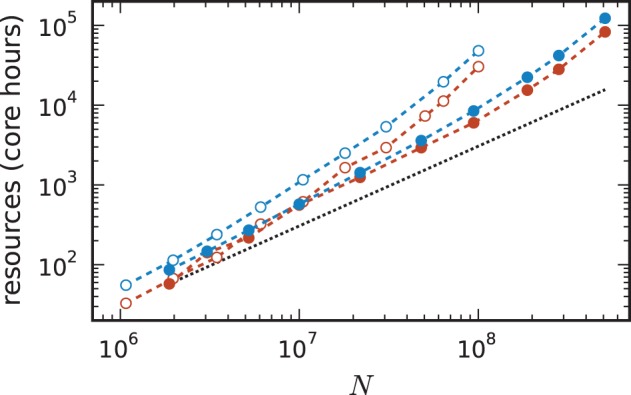
**Required resources to simulate a network of size *N***. Core hours are defined as *MT* × *T*_sim_ and do not account for idle cores of a node. Filled circles correspond to the 4g technology (see Figure 9) and open circles to the 3g technology (see Figure 7 for maximum network size and lower panel of Figure 8 for core hours). Results for K computer (red) and JUQUEEN (blue) using parameter set 1 (cf. Section 2.2). The dashed black line indicates the ideal case where the required resources increase with the same factor as the network size.

For a fair comparison we therefore show in Figure 11 the product of the runtime and the number of cores. The 4g technology shows a smaller slope of the dependence of the required resources on network size. This quantity is also of interest for the estimation of resources when planning research projects and applying for the required computation time as core hours are a commonly used unit in such documents. The duration of the simulation used for these benchmarks is 1 s of biological time. For longer times the core hours can be multiplied by the corresponding factor to obtain an estimate of the required resources. For example, given the resources of 10,000 core hours on the K computer we can perform a simulation of 1 s of biological time with about 7 · 10^7^ neurons using the 3g kernel but with more than twice as many neurons (1.5 · 10^8^) using the 4g kernel.

## 4. Discussion

The purpose of this study is not to gain neuroscientific insight, but to provide the technology to carry out full-scale neuronal network simulations at the resolution of neurons and synapses on presently available supercomputers. The work addresses the problem of efficiently representing a neuronal network on a supercomputer in a distributed manner, so that high performance is achieved while maintaining flexibility and generality. Starting from the existing simulation technology and our previous work on a mathematical model of the memory consumption (Kunkel et al., 2012b) of neural simulation codes, we employ and extend the analysis of memory consumption to expose a generic problem of a combinatorial nature. The problem can be summarized as follows: The number of interaction partners (synapses) of each element (neuron) is fixed by nature (about 10^4^), but the number of employed compute nodes may vary over many orders of magnitude, from a laptop (order 1) to a petascale supercomputer (order 10^5^). On supercomputers and for general connectivity of the network we inevitably face the problem of sparsity, where a neuron has a target on a given compute node only with small probability. Conversely, in a single core simulation, each neuron has all its targets on the very same core. Hence each end of this range requires different data structures for an effective representation of the synapses. On supercomputers this data structure must be distributed to cater for the memory demands of full-scale network simulations at cellular resolution. In the limit where each neuron only has none or a single synapse on a given machine the present data structure (Helias et al., 2012) exhibits a two-fold redundancy: the amount is fixed (just 1) and the type of the synapse is unique. We here describe a novel data structure that allows full flexibility if a neuron has multiple targets on a local machine, but that collapses to a static data structure in the limit of a single target. We also redesigned the underlying synapse data structure and the connection algorithm to reduce the memory footprint of a synapse, and finally developed a new data structure to contain local nodes along with a corresponding look-up algorithm exploiting the regularity in assigning neurons to machines.

The network model and the corresponding model of memory consumption presented here constitute a worst case scenario for memory consumption and communication in the sense that there is no structure which can be exploited to efficiently map the topology of the network to the topology of the computer. Moreover, in NEST neurons are distributed across virtual processes in a round-robin fashion, which allows for efficient static load balancing but prevents taking into account network structure. Kunkel et al. (2012a) investigate the case where a fraction of the incoming synapses has a local origin such as, for example, in a cortical network. The analysis shows that representing locally connected substructures on a subset of the available compute nodes reduces memory consumption. However, the advantage of such a topology-driven distribution scheme over a round-robin scheme diminishes with the adaptive connection infrastructure of the 4g simulation kernel as the novel data structures enable efficient storage of short target lists.

Simulation software on supercomputers often employs highly specialized and optimized code written in languages with a low degree of abstraction to maximally profit from machine-specific optimizations and optimizing compilers. We here meet the need for flexibility and generality with a different approach, employing features of a high-level object oriented language, such as metaprogramming and polymorphism as well as object-oriented design patterns to reduce the memory consumption, while maintaining flexibility, generality, and performance. Concretely, we use recursive C++ templates to formulate a container class with similar functionality as a vector but only one third of the overhead in the limit of only a few entries. An abstract base class provides a common interface to the data structure that allows nesting of containers whenever needed, e.g., to distinguish different synapse types. We swap out the polymorphism from synapse objects to a nested helper class to avoid memory overhead due to the virtual function pointer, but yet have polymorphic behavior at hand to implement a handshaking mechanism at connection setup. We formulate the connection framework in general terms as templates with the synapse type and the target identifier as template parameters. This separates the infrastructure from the neuroscientifically interesting part of the code, that represents the synaptic dynamics. This separation eases the extension of the simulator by new synapse types. Different implementations of the target pointer (full pointer, indirect indexed addressing) can be combined flexibly with the same code for a synapse by changing one additional template parameter. The templates hence enable code generation for synapses in different combinations with types fixed at compile time, so that the interesting, and user extensible code of synaptic dynamics exists only once.

The resulting data structures reduce the memory consumption on a supercomputer by up to a factor of four. Not only is the memory footprint smaller, but the new memory layout also decreases both the time to connect and the time to simulate a network of a given size. The latter increase in performance is likely due to more efficient use of the cache. The same network can hence be simulated with fewer resources (core hours), or reversely, the same amount of resources enable the simulation of around twice as large networks with the 4g kernel compared to NEST 2.2 (3g). Thus, computing time awarded on supercomputers is used more efficiently. Also, a typical simulation run on a laptop benefits from the presented optimizations: simulating the model used for all benchmarks in this paper with 11,250 neurons and 6000 incoming synapses per neuron (parameter set 2) on a single core for a biological time of 1 s takes 35.5 s with both NEST 2.2 (3g) and the 4g kernel while the memory consumptions significantly decrease from 5.24 GB for NEST 2.2 to 3.11 GB for the 4g kernel. As a result of these theory-guided improvements to memory structures, the observed dominance of overhead has been eliminated.

For a given size of the computer, the new data structures enable the simulation of networks of larger size. Employing the entire K computer with all 8 cores of each processor, the network filling the effectively available memory of 1.07 PB of RAM has 1.86 · 10^9^ neurons and 11.1 · 10^12^ synapses, each represented with a double precision synaptic weight and STDP dynamics. This is the largest simulation to date in terms of connectivity. It took 793.42 s to build the network and 2481.66 s to simulate 1 s of biological time. The new technology can exploit the full size of JUQUEEN to simulate a network of 1.08 · 10^9^ neurons with 6.5 · 10^12^ synapses, a network in the range of a cat's brain.

The technology described in these pages is general in the sense that it can be used in any neuronal simulator satisfying the following three constraints: (1) neurons are atomic and simulated as a whole on a single node of a parallel machine, (2) synapses are stored on the nodes on which the postsynaptic neurons reside, and (3) spikes are communicated globally using, for example, collective MPI communication. In an application where the communication scheme ensures that each machine only receives spikes from neurons with local targets, the sparse table is not required, however, additional infrastructure is needed on the presynaptic side. The C2 simulator (Ananthanarayanan and Modha, 2007), for example, employs such a directed communication; for a comparison of different spike exchange methods see Hines et al. (2011). In order to represent the connections between different compartments of a neuron distributed over several compute nodes, such as in the NEURON simulator (Hines et al., 2008) or the Neural Tissue Simulator (Kozloski and Wagner, 2011), the data structures presented here require major adaptation. In addition to the spike events also events mediating the instantaneous electrical interaction between compartments need to be communicated. If the number of coupled compartments is small compared to the number of compute nodes, we believe the metaprogramming technique to realize low-overhead vectors is still applicable. Related data structures may even be useful in other domains beyond neuroscience that face the problem of mapping a fixed number of heterogeneous interaction points on a number of CPU cores varying over many orders of magnitude. With exascale computers on the agenda, the ratio between interaction points and CPU cores is likely to drop further, increasing the sparsity of the problem.

Memory allocation can be improved beyond the solution presented here. In the limit of large numbers of compute nodes, we use a simple pool allocator for synapse objects that does not support reallocation of memory. In the limit where each neuron with high probability only has either zero or one target on a given machine this is not of practical concern, because target lists almost never grow beyond length one, so no reallocation is needed. In the intermediate regime, where target lists contain a few entries, an improved allocation scheme is beneficial. A drawback of the presented solution is that it requires the user to be aware of the simulation operating in the sparse regime. On a laptop, the allocator should be switched off, which is the default. In the regime of small and mid-size machines, an alternative to a pool allocator would be to determine the number of synapses ultimately generated in a preceding phase, such that the correct amount of memory can immediately be allocated. This approach, however, rules out the flexibility to implement structural plasticity and mechanisms of degeneration.

The benchmark information presented is required to apply for computation time grants, which typically request a proof of efficient use of the resources and a justification of the resources applied for. Strong scaling is one commonly requested measure. Our work shows that this measure is not relevant for all applications, in particular not for network simulations. The reason is that the typical neuroscientific use case of a supercomputer is close to the point of maximum filling, where the entire memory is used to represent the largest possible network on a given number of compute cores (see van Albada et al., 2014 for a discussion of further difficulties in assessing the performance of simulation codes for neuronal networks). The number of neurons per core is limited by the need to represent all synapses, rather than by the computation required to carry out the simulation. Reducing this number therefore further decreases the load of the CPUs. In strong scaling where the filling is systematically reduced, our benchmark simulations do not exhibit supra-linear scaling. Another measure occasionally required in applications for computation time is the number of executed floating point operations per second. For network models described here the absolute number of floating point operations in the code is small. The update of the neuronal state variables requires on the order of 10 floating point operations per time step and neuron. The majority of operations in network simulations as described here are related to random memory access caused by the delivery of the spiking events. Thus, without normalization for the fraction of floating point operations actually in the code, the relevance of the measure for neuronal network simulations is limited. In the extreme case of a code not using floating point numbers at all, say by the use of fixed point arithmetic or in case of a data base application, the floating point performance is exactly zero. Assessing the scalability of an application by weak scaling requires the problem size per machine node to be constant and in addition the problem to be representable in the memory available per node. Applications with computational overhead growing with machine size show the best weak scaling, if the problem size per node is maximized. In distributed neural simulations this overhead is dominated by communication. If memory overhead grows with machine or problem size (here: the employed sparse table), weak scaling requires the problem size to be chosen such that the problem still fits into memory at the largest machine size; at smaller machine sizes, there will hence be unused memory. In this study we employ maximum-filling scaling, using the maximal problem size per node at each machine size. For the 4g kernel this measure is very close to a weak scaling, as shown in Figure 9 as the memory overhead is low. For the 3g kernel, however, the problem size depends sub-linearly on the machine size (Figure 7). Therefore, in the weak scaling scenario the scalability of the 3g kernel is worse than the one of the 4g kernel just because the smaller problem size reduces the workload per core such that the contribution of communication overhead to the total run time is more severe. We use maximum-filling scaling here for two reasons: (1) we aim at a harder comparison of the 4g kernel vs. the 3g kernel and (2) we are interested in a measure of the minimal resources (core hours) required to simulate a given neuroscientific problem. For our benchmark network this minimum is achieved at the point of maximum filling (data not shown). Deeper insight into the effectiveness the algorithm achieves in operating on the entities of the simulation, in our case the neurons, may be gained by normalizing the simulation times obtained for maximum filling by the workload, in our case neurons per core.

Computing time is typically accounted in units of the product of wall-clock time and either compute nodes or CPU cores. The benchmarks presented in this manuscript employ 8 cores per compute node, even though more cores are available per node on JUQUEEN. An accounting system measuring resources by the product of nodes and hours will therefore punish a memory intense application that, due to the small workload per core, does not employ all cores of a node. Nevertheless, assignment of the entire node to a single application is of course required due to the lack of memory for other applications.

When a supercomputer and not the personal workstation is the target of a new neuronal network model some further difficulties arise. Below is a brief account of our experience and the strategies we developed so far to cope with them. On novel supercomputing platforms, such as the K computer, provenance tracking of the simulation environment is particularly challenging, because of the co-development of the hardware, the operating system components, and the application software. Additional robustness of the workflow is required because the researcher maintaining the simulation script may not be the person submitting the jobs and collecting the results. Performing simulations on supercomputers is time consuming due to the long queuing time and errors in a simulation script can often only be detected by actually running the simulation. Moreover, finding the maximum network size that consumes all memory on a machine in an iterative trial-and-error fashion can consume considerable amounts of resources. We here followed a two step procedure, first obtaining from the memory-usage model a prediction of the maximum number of neurons fitting on a given portion of the machine, and then running a series of “dry runs,” executed only on a single node and mimicking the existence of the remainder of the network (see Section 2.6). An extension of the dry-run feature including the simulation phase is desirable, because it would allow the scientist to uncover errors in the simulation script during and after the simulation phase prior to the execution of the actual simulation and also because the memory consumption during simulation may depend on the actual dynamics due to the allocation of communication buffers. Moreover, the dynamics may depend on the network size, so that test runs of a reduced problem on a smaller machine do not result in an accurate estimate of the resources. Capturing such effects by a dry run, however, requires a problem-specific mathematical model of network activity, the absence of which may have motivated the simulation project in the first place.

Current simulation technology is still hampered by the bottleneck of writing the simulated data generated in parallel on many processors to disk. The employed standard file I/O is easily overburdened by on the order of 100,000 processors in parallel opening files. More research is needed on how to include general solutions developed for parallel I/O into the neuronal simulation technology or to develop alternative solutions collecting the data on a small number of processors before writing to disk.

The simulation times achieved by the current technology are sufficiently short to study dynamical features of neuronal networks on the time scale of seconds of biological time. Synaptic plasticity, however, requires biological times of minutes to hours, leading to wall-clock times in the range of 100–10,000 h, which are typically beyond the resources available to a neuroscientist and impractical for exploratory research.

We present a viable solution to efficiently use the largest supercomputers available today for neuronal simulations. The exascale generation of supercomputers with even larger numbers of cores, however, is likely to require a new architecture. The mathematical analysis of memory consumption presented in the current work exposes the sparse table of neurons with local targets which has been the backbone of the communication algorithm since the first distributed neuronal simulations as the limiting component; it grows proportional to the total number of neurons in the network. As for each neuron one bit must be stored, indicating whether or not the corresponding neuron has a target on the given machine, the growth of the sparse table will ultimately limit the scalability of the software. This tendency can already be observed in Figure 9. The need for the sparse table arises from the collective communication scheme employed so far. The emission of an action potential by a neuron is communicated to all other compute nodes. These, in turn, decide with the help of the sparse table whether or not the sending neuron has a local target. Collective communication is a good implementation on the current petascale machines: Since there are around 2000 neurons simulated per core with 10,000 incoming synapses each, the probability that, assuming a random network, at least one spike must be communicated from one core to another is close to one. However, as each neuron sends its spike to all other *MT* cores, but only has targets on at most *K* of them, per neuron at least *MT* − *K* spikes must be discarded on the other receiving cores.

Omitting the sparse table altogether requires directed communication to only those machines that harbor a target of the sending neuron. Two consequences arise for the required data structures. First, there needs to be a representation of the outgoing connections on the machine of the sending neuron, namely for each neuron one must know the nodes on which the neuron has targets, as already described in Morrison et al. (2005b). Second, the receiving machine needs a map from the id of the sending neuron to a list of its targets. Future work is required to find memory efficient implementations for these two data structures, algorithms to instantiate them during network setup, and appropriate communication methods. Such a framework will still benefit from the adaptive data structures presented in the current work for representing the actual synapses.

The simulation technology presented in this manuscript enables the scientific community to make full use of petascale supercomputers for neuroscientific research.

### Conflict of interest statement

The authors declare that the research was conducted in the absence of any commercial or financial relationships that could be construed as a potential conflict of interest.

## Appendix

**Algorithm 1:** **Recursive definition of homogeneous containers implementing low-overhead vector for small numbers of elements**. The *K* data elements (synapse objects) of template type connectionT are stored in a C-style array (line 10) and hence consume *K* · sizeof(connectionT) Bytes. 8 B of additional overhead are due to the virtual function table (vtable) pointer inherited from the abstract base class ConnectorBase (see also Figure 4). The first template definition (line 6) serves as the recursion continuation step with the number *K* of stored elements as the recurrence parameter. The method push_back is inherited from the abstract base class vector_like. For template arguments *K* < *K*_cutoff_ push_back recursively instantiates a container of size *K* + 1 (line 27) at compile time, joins the previously stored contents with the new element and destroys itself (line 31). The new container is returned to the caller and is stored in the adaptive data structure (see also Algorithm 2). Recursion termination is achieved by providing a template specialization for *K*_cutoff_ (line 39), which uses a std::vector<connectionT> to store the data elements.*K*_cutoff_ = 3 is a preprocessor define fixed at compile time.

1 // *inheritance from abstract base class*
2 // *1. vector_like: to provide push_back as common interface*
3 // *2. ConnectorBase: to have common base class type for all containers*
4
5 // *recursion step*
6 **template**<**int** K, **typename** connectionT>
7 **struct** HomConnector
8 : **public** vector_like <connectionT>
9 {
10 connectionT C_[K];
11
12 HomConnector(**const** HomConnector<K−1, connectionT> & Cm1,
13 **const** connectionT & c)
14 : syn_id_(Cm1.syn_id_)
15 {
16 **for** (**int** i=0; i<K−1; i++)
17 C_[i] = Cm1.C_[i];
18 C_[K−1] = c;
19 }
20
21 // *pushback: recursion step to next container*
22 HomConnector <K+1, connectionT> & push_back(**const** connectionT & c)
23 {
24 //*pass on contents and*
25 // *recursively instantiate container for K+1 elements*
26 // *use pool allocator, pass (*this, c) as args to constructor*
27 HomConnector<K+1, connectionT> *newconn =
28 allocate <HomConnector<K+1, ConnectionT> >(***this**, c);
29
30 // *delete this instance*
31 **delete this**;
32
33 // *return container of next size*
34 **return** *newconn;
35 }
36 };
37
38 // *recursion termination by template specialization*
39 **template**<**typename** connectionT>
40 **struct** HomConnector<K_cutoff, connectionT>
41 : **public** vector_like <connectionT>
42 {
43 std::vector<connectionT> C_;
44 …
45 HomConnector<K_cutoff, connectionT> & push_back(**const** connectionT & c)
46 {
47 C_.push_back(c);
48 **return** ***this**;
49 }
50 };

**Algorithm 2:** **Algorithm to create new connections using adaptive data containers**. The algorithm is implemented in the method add_connection() of ConnectorModel (see also Figure 4). The template function allocate<T> invokes the custom pool allocator, providing for each thread memory from a separate pool. The different branches of the algorithm marked by Case 0-3 refer to the description in the main text.

1 ConnectorBase* add_connection(Node& tgt, Node& src,
2 ConnectorBase* conn, ConnectionT& c):
3
4 // *source neuron does not yet have any local targets (Case 0)*
5 **if** conn == 0:
6 c.check_connection(t_lastspike)
7 conn ← allocate <HomConnector<1, ConnectionT> >(c)
8
9 // *source neuron has at least one local target*
10 **else**:
11 c.check_connection(t_lastspike(*conn).get_t_lastspike())
12
13 // *all existing local synapses are of the same type (Case 1,2)*
14 **if** (*conn).homogeneous_model():
15
16 // *new synapse is of same type as existing ones (stay in Case 1,2)*
17 **if** (*conn).get_syn_id() == c.get_syn_id():
18 hom_vec ← **static_cast**<vector_like<ConnectionT> * >(conn)
19 conn ← &(*hom_vec).push_back(c)
20
21 // *new synapse is of different type than existing ones (switch to Case 3)*
22 **else**:
23 het_conn ← allocate <HetConnector>()
24 (*het_conn).push_back(conn)
25 hom_conn ← allocate<HomConnector<1, ConnectionT> >(c)
26 (*het_conn).push_back(hom_conn)
27 conn ← het_conn
28
29 // *different types of local synapses exist (Case 3)*
30 **else**:
31 het_conn ← **static_cast** <HetConnector>(conn)
32 **foreach** h_conn **in** het_conn:
33
34 // *at least one local synapse of this type already exists*
35 **if** (*h_conn).get_syn_id() == c.get_syn_id():
36 hom_conn ← **static_cast**<HomConnector<ConnectionT> * >(h_conn)
37 hom_conn ← &(*hom_conn).push_back(c)
38 found ← **true**
39 **break**
40
41 // *local synapses of this type do not yet exist*
42 **if not** found:
43 hom_conn ← allocate <HomConnector<1, ConnectionT> >(c)
44 (*het_conn).push_back(hom_conn)
45
46 **return** conn

**Algorithm 3:** get_node_by_gid() in SparseNodeArray returns 0 for non-local nodes and the pointer to the requested node for local nodes.

1 Node* get_node_by_gid(index gid):
2
3 **if** gid > max_gid:
4 **throw** UnknownNode() // *node does not exist*
5
6 **if** gid == 0:
7 **return** local_nodes[0].node // *root node*
8
9 **if not** (local_min_gid ≤ gid ≤ local_max_gid):
10 **return** 0 // *node not local*
11
12 // *estimate location in sparse node array*
13 idx ← floor(1 + alpha * (gid − local_min_gid))
14
15 // *search to the left*
16 **while** 0 < idx **and** gid < local_nodes[idx].gid:
17 idx ← idx−1
18
19 // *search to the right*
20 **while** idx < local_nodes.size **and** local_nodes[idx].gid < gid:
21 idx ← idx+1
22
23 **if** idx < local_nodes.size **and** local_nodes[idx].gid == gid:
24 **return** local_nodes[idx].node // *local node found*
25 **else**:
26 **return** 0 // *node not local*
